# **Human burials indicate climate-mediated shifts in South African agriculturalist demography after 2000 years ago**

**DOI:** 10.1038/s41598-025-05471-6

**Published:** 2025-07-01

**Authors:** Emma Loftus, Maryna Steyn, Marlize Lombard, Brian M. Chase

**Affiliations:** 1 Institut für Vor- und Frühgeschichtliche Archäologie, Ludwig-Maximilians-Universität München, Geschwister-Scholl Platz 1, Munich, 80539 Germany; 2 Human Variation and Identification Research Unit, School of Anatomical Sciences, Faculty of Health Sciences, University of the Witwatersrand, Johannesburg, South Africa; 3 Palaeo-Research Institute, University of Johannesburg, Auckland Park, P.O. Box 524, ZA-2006 Johannesburg, South Africa; 4 Institut des Sciences de l’Evolution–Montpellier, University of Montpellier, Centre National de la Recherche Scientifique, EPHE, IRD, Montpellier, France; 5 Department of Environmental and Geographical Science, University of Cape Town, South Lane, Rondebosch, South Africa

**Keywords:** South Africa, Iron age, Human burials, Demography, Climate change, Palaeoclimate, Archaeology

## Abstract

South Africa’s Iron Age (c. 250 CE – 1850 CE) was a period of socio-economic transitions. With the spread of Bantu-speaking peoples from western Africa, the region saw the development of settled agriculturalist societies, the rise of complex chiefdoms, and the development of early states such as Mapungubwe. Questions about how settlements and demographics were influenced by environmental factors are central to the study of this period. Climatic conditions directly impact carrying capacity and agricultural productivity. It is therefore likely that climate variability was an important factor in the success of farming communities, determining food security, population movements, and settlement expansions or abandonments. In this paper we focus on northeastern South Africa and employ the frequency of archaeological human burials and highly resolved palaeoclimate records to assess the relationship between population dynamics and climate change. This reveals significant subregional variability in both human burial frequency and climate change across the subcontinent. Within the subregions, burial frequency shows a positive correlation with humidity, indicating that climatic factors played a pivotal role in shaping the pattern and size of settlements during this period.

**Supplementary Information:**

The online version contains supplementary material available at 10.1038/s41598-025-05471-6.

## Introduction

Archaeological finds of human remains represent one of the best direct sources of evidence for understanding the past at both the scale of an individual human life, and in population aggregates. Human remains provide otherwise impossible-to-recover information about a person’s lived experiences, and collectively speak to wider questions of demography, health and social behaviours^1- 8^. While many studies have investigated human remains to infer adaptations of individuals and groups to the environment ^9-12^, less has been done in a broader context to look at the changes in the burial record in aggregate, particularly in southern Africa^13^.

The South African burial record of agricultural societies since 250 CE (known as the Iron Age in the local archaeological context) is in many ways partial and poorly documented. Early excavations, such as those on the Mapungubwe landscape, beginning in the 1930s, saw the removal of large numbers of human remains from their graves with poor standards of recording and documentation^14^. Even less information is available from other regions in southern Africa, limiting comparisons with spatiotemporally contiguous societies across modern political boundaries (e.g. Zimbabwe, Mozambique, Botswana). Descendent communities are also increasingly voicing objections against research that necessitates destructive sampling, and advocate for the repatriation of such remains from institutional repositories for reburial close to the original settlements. The methods presented in this paper therefore respond to a growing awareness of, and responsiveness towards, such ethical concerns, by offering a broader, synthetic perspective without the necessity for further destructive sampling or skeletal analysis. This is particularly cogent in contexts with descendant communities present, who have diverse and conflicting perspectives about the value and necessity of archaeological/scientific research, set against the sanctity of burials and human remains.

Recently, a rich database was compiled of dated human remains from the South African Holocene^15^ (see also^16^ for a summary of South African human skeletal repositories). These remains relate to hunter-gatherer/forager (throughout the Holocene until a few hundred years ago), pastoralist (since c.2000 years ago) and agriculturalist (since c.1700 years ago) archaeological contexts. Preliminary analyses of these data showed some marked spatio-temporal fluctuations in the numbers of both forager and agriculturalist burials^15^. While it was considered possible that these fluctuations corresponded broadly with major socio-economic and climatic shifts, spatial variability in the timing of such shifts across the region has limited any conclusions about numbers of human remains through time at the regional scale.

Further, while the number of radiocarbon dates from the archaeological record overall is heavily biased towards the last two millennia (c.50% of the 2500 dates in the Southern African Radiocarbon Database^17^, the number of directly dated *burials* from agriculturalist contexts is far outnumbered by those from forager sites^15^. Direct dating is essential for single and isolated contexts (e.g. dune burials, or rockshelter deposits) from which forager remains were often removed, but the remains of agriculturalists are typically recovered from settlements, many of which are single-phase occupations. Such burials are often dated by their associations with ceramic traditions that discriminate assemblages at the scale of a few hundred years ^18^, and destructive analyses of such skeletons have more ethical considerations.

This situation poses a challenge for studies that seek to reconstruct changing frequencies of agriculturalist burials through time, as there are many more undated sets of human remains than there are directly dated ones. Moreover, the assemblage of direct ages produced in Loftus et al.^15^ (see Fig. 2) suggested a marked decline in the distribution of agriculturalist dated remains between c.1050–1250 CE. Yet, this decline overlaps with a period of early state formation referred to by Huffman^18^ as the Middle Iron Age (c.900–1300 CE), an archaeological phase that generally relates to sites in the Shashe-Limpopo basin along the South African-Zimbabwean-Botswanan border. In fact, substantial numbers of human remains were excavated from sites dating to this period, such as Schroda and Mapungubwe, with more than 100 individuals from K2 (also known as Bambandyanalo) alone^19^, but they were relatively dated by ceramic traditions and associated radiocarbon dates rather than being directly dated. Thus, the reconstructed radiocarbon date curve does not reflect the true frequency of burials over this interval, pointing towards obvious biases in the radiocarbon record, and prompting the development of a method that better reflects the underlying archaeological record.

Only a small proportion (< 10%) of South Africa today is considered suitable for non-irrigated (or “small-scale”) crop farming, with limited, erratic precipitation and generally high aridity cited as principal limiting factors^20,21^. Yet, the distribution and reliability of precipitation differs markedly across the area settled by crop agriculturalists (i.e. “Iron Age farmers”), from subtropical and temperate regions in the south-east (mean annual precipitation [MAP] > 800 mm) to semi-arid and arid environments in the central, northern and north-eastern parts of the country. We thus anticipate variable responses to past climatic shifts. In arid and semi-arid regions, even small fluctuations in moisture availability would have significantly affected primary productivity and the suitability of such landscapes for agriculture and settlement. Conversely, settlement should be less affected by negative deviations in precipitation in the south-eastern sub-humid regions. This is supported by contemporary observations of drought hazard, which indicate the highest drought risk for agriculture in the northern and north-western parts of the country, while the lowest risk is in the south-east^22^.

In this paper we address the biases in the dated archaeological record using a method of combining both direct and indirect age estimates for the reconstruction of burial frequencies through time. For this, age estimates are generated for each undated set of remains based on the associated site dates and simulating a radiocarbon age within this age estimate, creating a more comprehensive dataset of archaeological human remains from agriculturalist contexts (compared to^15^). Through a detailed consideration of South Africa’s complex palaeoclimatic history, these data are divided into subregional groups according to topographic boundaries. For each subregion, we have produced summary age models with overlapping sets of both direct and indirect simulated ages, according to a confidence categorisation assigned to each age. These models are analysed using archaeological records and palaeoclimatic data that best reflect climate change in each subregion, with the goal of identifying spatial patterns in human burial behaviours and their correlation with climate variation, particularly precipitation as a limiting factor for crop agriculture, in north-eastern South Africa over the past 1500 years. While this attempt to link burial frequencies (as a proxy for population size) to climatic indicators has limitations, as discussed later, it provides a new approach to estimating population shifts in southern Africa.

## Results and interpretation

### Frequencies of human remains from agriculturalist archaeological sites through time

**Fig. 1:**
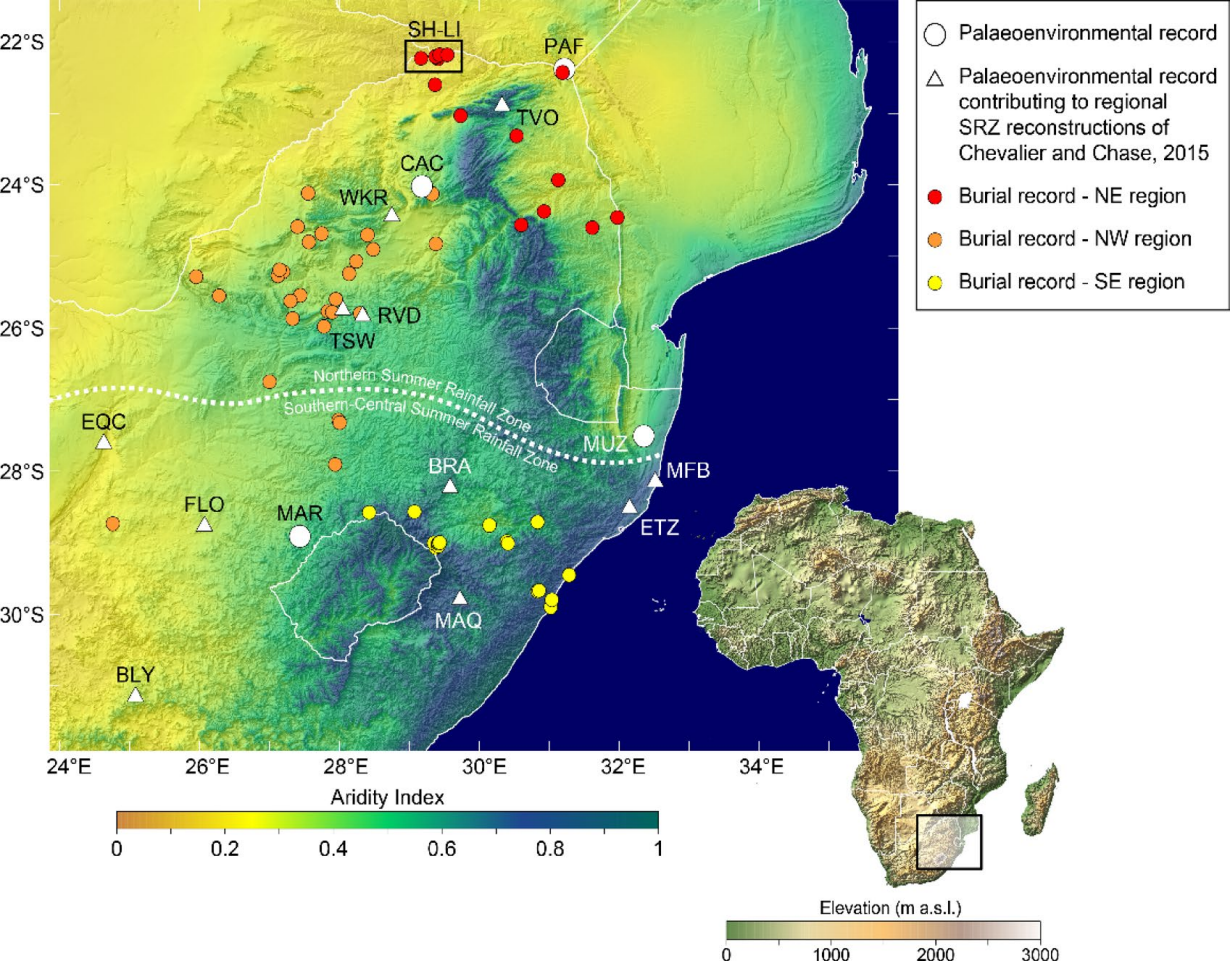
Map of study region indicating burial sites divided into subregions (red, orange and yellow circles) and relevant palaeoenvironmental records from the region. White circles denote the high-resolution records selected for detailed comparison: Pafuri (PAF;^23^), Cold Air Cave (CAC;^24^), Marakabi (MAR;^25^) and Lake Muzi (MUZ;^26^). White triangles denote the sites included in the regional temperature and precipitation reconstructions of Chevalier and Chase^27^ for the summer rainfall zone (SRZ): (Tate Vondo (TVO;^28^), Wonderkrater (WKR;^29^), Tswaing Crater (TSW;^30,31^) and Rietvlei (RVD;^32^), and southern-central summer rainfall zone: Braamhoek (BRA;^33^), Florisbad (FLO;^34^), Equus Cave (EQC;^35^*)*, Blydefontein (BLY;^36^), Mahwaqa Mountain (MAQ;^37^) Lake Eteza (ETZ;^38^) and Mfabeni (MFB;^39^). This figure was produced using Surfer by Golden Software (v. 27; https://www.goldensoftware.com/products/surfer/): the Aridity Index data is from Trabucco and Zomer^40^ while the digital elevation model is from Yamazaki et al.^41^. The Shashe-Limpopo Basin (SH-LI) is indicated in the northern portion of the map.

To evaluate the role of geographic or climatic factors in the frequency of agriculturalist human burials through time, we have defined three subregions based on topographic boundaries (Fig. 1). The largest group (*N* = 181), in the northeast of the country (NE), includes sites in the archaeologically rich Shashe-Limpopo Basin (also known as the Mapungubwe landscape) and the Mpumalanga Lowveld. The north-western group (NW; *N* = 93) includes those sites in the Highveld west of the central and northern ranges of the Great Escarpment and Drakensberg range. Finally, the smallest group (*N* = 40) includes remains from sites in and to the southeast of the high mountain areas, largely in the current KwaZulu-Natal province (SE). These regions reflect the distribution of most South African Iron Age sites and associated archaeological research^18^.

**Fig. 2:**
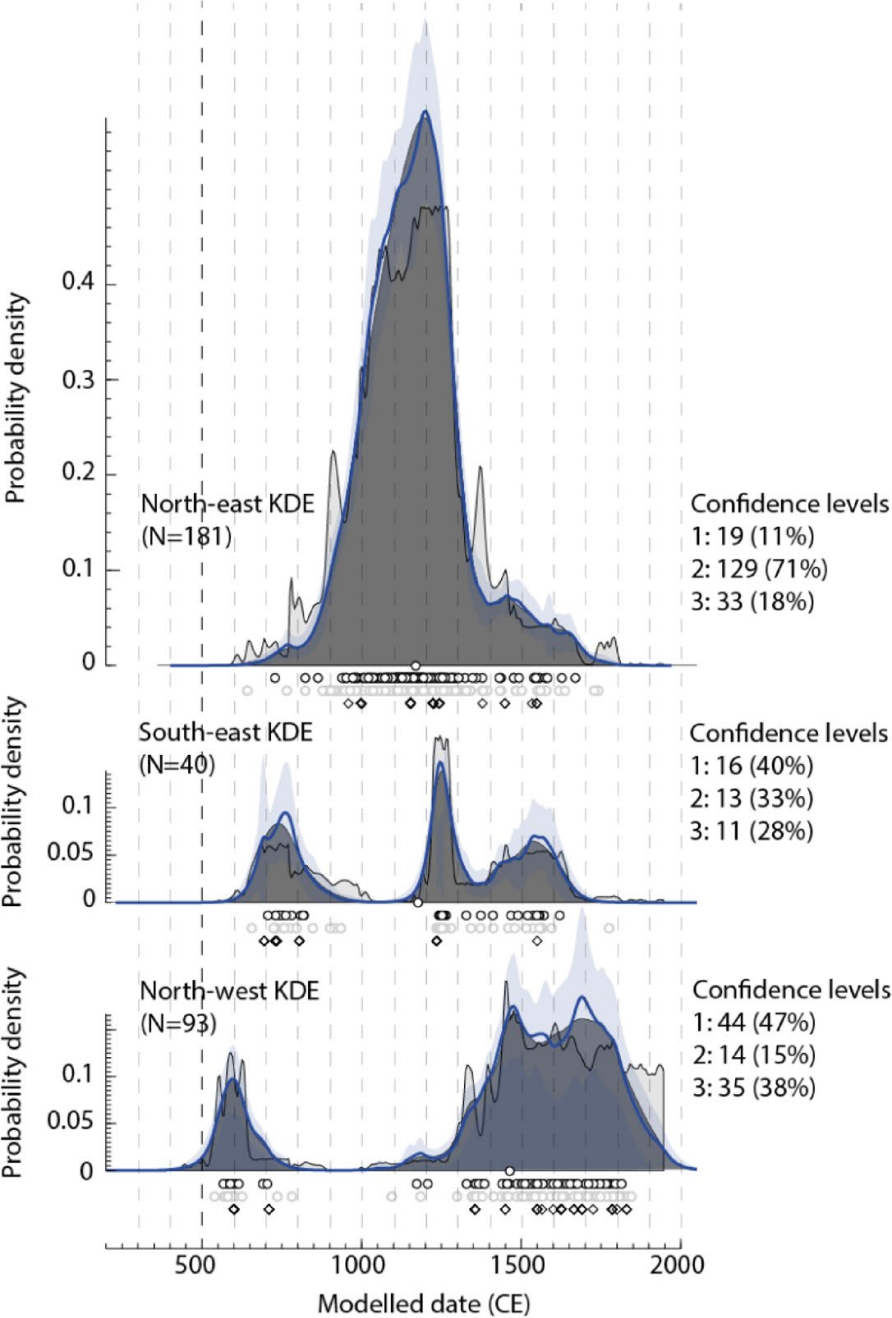
Kernel Density Estimation models of radiocarbon dates and simulated ages (as specified by the diamonds) for human remains separated by regions (see Fig. 1). Also shown are the numbers of human remains ages by confidence category reflecting the quality of the dating information (see Methods), with the percentages for each subset.

Dating information was assessed and structured according to data quality and Kernel Density Estimation (KDE) methods were used to summarise the distributions of the ages for each region (Fig. 2, see Methods). The burial age KDE distributions from these regions exhibit significant differences between each subregion (Fig. 2), supporting spatial subdivision of the data.

### Climate shifts and regional distributions of burials through time

To define a context of environmental change for the variability observed in the burial data, we have compiled relevant records from the region. Reconstructions of precipitation, temperature^27^ and aridity^42^ indicate long-term trends of progressively drier conditions in South Africa’s summer rainfall zone (*sensu*^43^) over the last 1500 years. At broad regional scales, precipitation in the southern and central summer rainfall zone appears to have declined markedly from 500 CE until 1750 CE, after which a sharp increase is reconstructed until the end of the record at 1950 CE^27^. A similar reconstruction for the northern summer rainfall zone indicates higher variability of precipitation, but a long-term decline in precipitation overall. Based on this suite of reconstructions, across the summer rainfall zone, increasingly arid conditions may relate to generally increasing temperatures, particularly since 950 CE^27,42^.

**Fig. 3:**
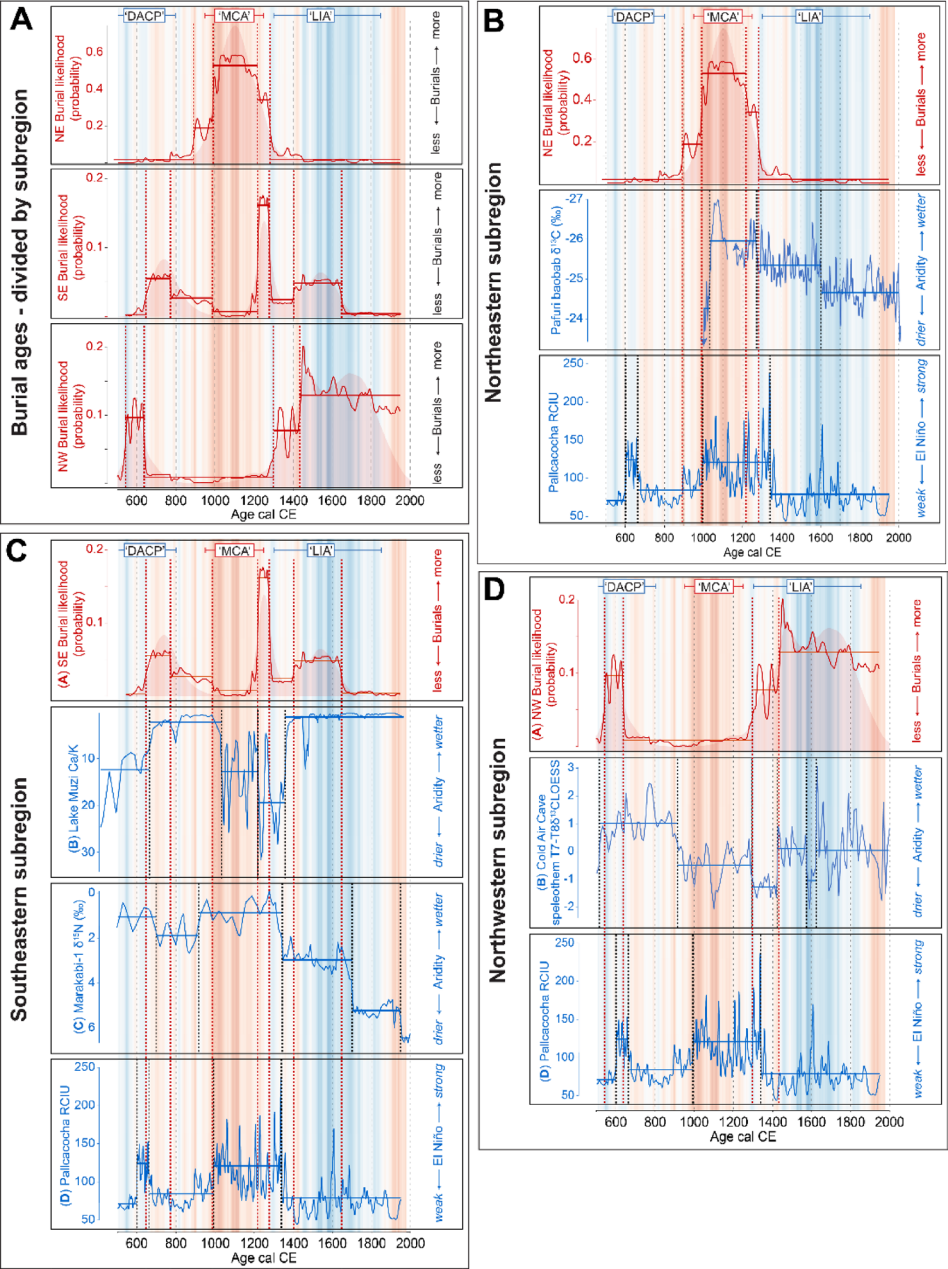
Aggregate models of the human remains data and palaeoenvironmental records divided into NE, NW and SE subregions. Background shading in each pane reflects warmer (red) and cooler (blue) temperature anomalies in the Northern Hemisphere temperature reconstruction of Moberg et al.^48^. The timing of the Dark Ages Cold Period (DACP), Medieval Climate Anomaly (MCA) and Little Ice Age (LIA) are shown. Dashed lines indicate where the mean of each signal changes most significantly, with horizontal lines describing the mean value of each segment (change-point analysis;^26^). Palaeoenvironmental records include Pafuri^23^, Lake Muzi^24^, Marakabi^49^, Cold Air Cave^50^, and Laguna Pallcacocha red colour intensity units (RCIU)^51^.

At the scale of the subregions identified, more highly resolved records of late Holocene climate change are available from the Cold Air Cave speleothems^24,44–46^, the Marakabi-1 rock hyrax midden^25^, Lake Muzi sediment record^26^ and the Pafuri tree ring record^23^. When considered together, these records indicate a more complex history of regional climate dynamics over the last 1500 years (Fig. 3).

The primary pattern defined by the burial data appears to be organised in three general phases that are broadly consistent with periods of relatively warm and cool conditions at the global scale, the Dark Ages Cold Period (400–800 CE), Medieval Climate Anomaly (950–1250 CE) and Little Ice Age (1300–1850 CE)^26,48,52,53^. The response to these periods is variable between the subregions, with an increase in burials observed in the northeastern subregion during the Medieval Climate Anomaly while fewer burials are observed in the northwest and southeastern subregions (Fig. 3A). In the NE subregion, δ^13^C data from the Pafuri baobab trees suggest that the Medieval Climate Anomaly was relatively humid^23^; Fig. 3B). The subsequent trend towards drier conditions can be statistically divided using change-point analysis^24^ into two significant phases with transitions at c.1300 CE and c.1600 CE, similar in timing to decreases in burial frequency.

To the southeast, the patterns expressed by both the burial data and the Lake Muzi geochemical data^26^ stand in marked contrast to the northeastern subregion. With the exception of a marked but discrete peak in burial ages at the end of the Medieval Climate Anomaly, there are few burials recorded from this period. Rather, the late Dark Ages Cold Period and Little Ice Age see increases in burials concurrent with phases of increased humidity at Lake Muzi (Fig. 3C). It is perhaps worth noting that this subregion may be limited to the region between the coast and the Great Escarpment, as δ^15^N data from the Marakabi hyrax midden site^54^ indicate an opposing trend to that which is observed at coastal sites (Fig. 3C).

The NW subregion is clearly characterised by increases in burial records during the Dark Ages Cold Period and Little Ice Age, with few burials observed during the Medieval Climate Anomaly (Fig. 3D). This pattern - largely the inverse of that observed in the northeastern subregion - is again broadly consistent with regional palaeoclimate proxies (δ^13^C data from the Cold Air Cave T8 speleothem^49^), indicating a positive correlation between burials and humidity.

In terms of drivers of long-term climate change, El Niño-Southern Oscillation (ENSO) is often cited as being a dominant factor in eastern South Africa, with increased ENSO activity resulting in drier conditions and consequently decreased crop agriculture yields and pasture productivity (e.g^55,56^). Proxies used to infer ENSO activity, however, exhibit significant differences of the timing and amplitude of ENSO activity (cf^26,49,57^). Here, we employ the often-cited Laguna Pallcacocha record^58^ to facilitate comparisons with other relevant South African studies^49,56^. As with all putative ENSO proxies^26^, the fidelity of the Pallcacocha record as an ENSO indicator has been questioned^24^. Nevertheless, it does describe a pattern of variability that broadly aligns with that of the palaeoclimate proxy records cited here, albeit with distinct regional responses (Fig. 3). The phase of increased ENSO activity inferred from the Pallcacocha record^59^ and corresponding with the Medieval Climate Anomaly is marked by increased aridity in KwaZulu-Natal at Lake Muzi^60^, and in the northwestern subregion according to interpretations of the δ^13^C data from Cold Air Cave^25^. This is consistent with the observation that ENSO events are associated with decreases in rainfall in South Africa’s summer rainfall zone^60,61^. However, in the NE subregion at Pafuri^23^ and westward of the Escarpment at Marakabi^63^, an inverse response is observed, with increased ENSO activity inferred from the Pallcacocha record corresponding with more humid conditions. This suggests that a more complex range of drivers and responses is required to understand the factors determining climate variability in the region.

### The archaeological context for changes in burial frequency

Given the taphonomic and cultural factors that can affect the preservation and recovery of human remains in the archaeological record, it is evident that numbers of burials may not reflect demography directly (see Loftus and Pfeiffer^64^ for a southern African example where changing burial frequency is argued to reflect non-demographic processes). That said, the patterns reflected here are broadly consistent with demographic explanations. First, at least some of the burial practices observed by these societies are believed to have considerable antiquity (i.e. infant pot burials, adult burials in the central kraal)^62,63^, and there is no evidence for a major change in burial practices throughout the period.

Secondly, the distribution of dated burials in the north-east is consistent with palaeo-ecological understandings of the suitability of the region for crop agriculture. The archaeological sequence of the Shashe-Limpopo Basin (in the NE subregion, Fig. 1) is one of the best documented for the Iron Age in southern Africa. Located in what is today an arid region adjacent to the South African-Zimbabwean-Botswanan border^63^, the comparatively fertile floodplains alongside the Limpopo River attracted the first substantial communities of farmers to the region in the 9th century CE, near the start of the Medieval Climate Anomaly^64,65^. Archaeological evidence supports the cultivation of sorghum, millet and other crops, while large herds of cattle were kept at the height of the occupation of K2 and Mapungubwe^64^. The productive floodplain grasslands likely also supported large herds of elephants^66^, and Zhizo-period communities such as that based at Schroda became moderately wealthy through the establishment of an ivory trade into Indian Ocean networks. At the start of the 2nd millennium CE, however, these Zhizo groups were displaced (or subjugated: see^67,68^) by so-called Leopard’s Kopje peoples, who established a major settlement at K2. This occupation coincides with the start of a multi-century episode of generally higher rainfall - as indicated by the Pafuri baobab δ^13^C record^23^; Fig. 3B) - and the Leopard’s Kopje population benefitted from the improved agricultural conditions for cereals and cattle grazing along the margins of the floodplain wetlands.

As populations and social complexity increased, the centre of power moved from K2 and a new capital was formed nearby on the Mapungubwe Hill c.1220 CE. This settlement history appears to be reflected in the model of dated burials for the NE subregion, with a steady increase in burials beginning c.900 CE and seeming to accelerate c.1200 CE. Importantly, palaeodemographic evidence from the age distribution of buried individuals (especially from K2) indicates a rapidly growing population over this sequence with an overrepresentation of infant and juvenile burials (e.g^70^), further attesting to favourable agricultural conditions. The role of climatic shifts in the abandonment of Mapungubwe has been debated extensively^64,71,72^, but Huffman^74^ proposed that, given large populations at the time and their dependence on the seasonal flooding, even a relatively minor drought could have sufficiently disrupted the food and fuel supply, undermining royal legitimacy. Certainly, the site’s relatively sudden abandonment c.1300 CE is coincident with a marked decline in burials and a shift towards more arid conditions inferred from the Pafuri baobab δ^13^C record at the end of the Medieval Climate Anomaly (Fig. 3B), suggesting that regional climatic conditions could have played an important role in the region’s settlement.

Intriguingly, the burial and climate records for the NW and SE subregions follow an opposite pattern, with very few recorded burials and relatively dry conditions in the period from c.800–1200 CE (Fig. 3C, D). The near total lack of burial data in the NW subregion for the several centuries spanning the Medieval Climatic Anomaly strongly suggests that the region was only sparsely settled by agriculturalists at this time, supported by an overall paucity of radiocarbon dates for farming sites here during this period^75^. While this pattern of settlement could be attributed to the dominance of Shashe-Limpopo Basin leaders in the regional political dynamics of the time, it does not preclude an underlying environmental control, which rendered the NE more suitable for agriculture and thus to the development of the kingship model in this subregion. Upon the apparent depopulation of the NE region at c.1300 CE, an increase in burials in the NW suggests that at least part of the population moved westwards, as well as into better-watered areas on the Zimbabwean escarpment to the north where they contributed to the rise of Great Zimbabwe^76^.

The burial frequency record from the sub-humid SE subregion is more limited and less certain (Fig. 2), but it appears to express broad similarities with the NW subregion, specifically, periods of increased burial frequencies in the first and second millenniums with peaks centred on c.750 CE, c.1250 CE and c.1550 CE and a major decline in burial frequency c.950–1200 CE spanning the Medieval Climatic Anomaly as well as the Early to Later Iron Age transition (c.1000 CE in this region^73^). As with the other subregions described, these increases in settlements and burial frequency are concurrent with phases of more humid conditions, as observed in this case in the Lake Muzi sediment record, which has been interpreted as reflecting ENSO-driven changes in precipitation in the region^74^. Yet, the relationship is not as marked as in the other two subregions. This likely reflects the limited sample size (but see Fig. 6) but may also reflect the less stringent constraints imposed by small to moderate declines in precipitation in this region.

## Conclusion

Numerous studies from contexts around the world have explored correlations between archaeological activity as reflected in radiocarbon dates and climatic change, to evaluate cultural change with respect to concepts such as societal collapse and resilience (e.g^74,75^). Such approaches are facilitated by the growing availability and (open-) accessibility of regional and global databases of spatially referenced radiocarbon dates. While there is widespread recognition of the potential biases and shortcomings inherent in such data and the statistical methods used to aggregate them (reviewed in^76^), the literature is increasingly sophisticated and capable of generating powerful insights into human-environmental dynamics (e.g.^77–80^). The flourishing utility of this field is doubtless made possible by an abundance of high-quality data in many parts of the world (e.g.^81^). This study employs a combination of direct and indirect age estimates for the reconstruction of human burial frequencies across the eastern parts of South Africa in order to maximise the potential of regional datasets and to facilitate more rigorous comparisons of the archaeological record of the region through time. The generation of calibrated burial date summary curves has allowed us to identify significant spatial and temporal variability in burial frequencies and enabled an analysis of the correlation between regional climatic shifts and socio-economic changes, that would not have been possible using directly dated burials alone.

Overall, across the eastern portion of South Africa, a correlation is observed between phases of increased agriculturalist burial frequencies and more humid conditions, particularly in the two subregions most vulnerable to small shifts in precipitation. Considered in the context of the regional archaeology, these phases are often associated with evidence for an increased farming population, suggesting clear links between environmental conditions, agriculturalist population size and burial frequency. While limitations in data availability and biases remain, this approach highlights the importance of interdisciplinary research in uncovering the complex interplay between environmental and human dynamics in the past. Such insights contribute to broader understandings of how past farming communities adapted to changing climates, offering valuable lessons for interpreting human resilience and vulnerability in diverse contexts.

## Methods (1396 words)

Similar to the Loftus et al.^16^ paper, this research is based on the Morris^82^ catalogue of South African Holocene human skeletons, which we gratefully acknowledge. From this list, updated catalogues provided by various museum curators, and various published resources, an initial list of all dated skeletons were made. This list was then expanded to also include well-dated archaeological sites with human remains associated with them.

Using this basic list, all skeletons from the sites they represent were then added. The focus was on providing a list of all individuals that were excavated, and not only skeletons that are housed in collections and are currently available for further study. It aimed to represent the number of individuals whose remains were recovered. For example, for the K2/Mapungubwe remains, the list from Steyn^83^ was included, but here all remains documented to have been excavated^83,84^ were included, even if some of the remains were never accessioned because they were reported to be too fragmentary. The catalogue (included here as S1) is thus based on the numbers of individuals represented by the remains, and not necessarily the currently available skeletons. Some of the details and numbers come from our own, recent reassessments of skeletons from various sites, e.g. Clarens^85^ and Cathkin Peak^86^.

Age category and sex were recorded where known from the literature, and references for each site added. Although not all archaeologists will agree with the division of Huffman^88^ of Early Iron Age (100–900 CE), Middle Iron Age (900–1300 CE) and Later Iron Age (1300–1840 CE), it was retained here as it provides a widely-used temporal framework for agriculturalist societies over the last two millennia.

This dataset comprises 314 individuals, from 60 archaeological sites and curated across 10 institutional repositories, including reburial facilities. This is fewer than recorded by Baliso et al.^86^, as they included all remains in collections, regardless of whether they were firmly contextualized or not. The dating information ranged from a direct date on the human remains themselves through to a contextual age based on associated radiocarbon dates from the archaeological site (provided separately in the SOM file). In order to refine these contextual ages, the radiocarbon dates from each site (typically on charcoal) were calibrated and modelled using a *KDE_Plot* function in OxCal to produce an estimated calendar age for the settlement occupations related to the burials (given as µ ± σ in the SOM). This conservative estimate is incorporated into the KDE aggregate burial models using an *R_Simulate* command in OxCal, which generates a simulated radiocarbon measurement that matches the specified calendar age range.

At this point, the dating information for each record was sorted into one of three categories reflecting the confidence or quality of the date. Direct dates, or radiocarbon dates from a very closely associated sample (i.e. a charcoal sample or burial in the same pit) were given the highest confidence level (*N* = 79). This group differs slightly from that published in Loftus et al.^87^ as it includes more individuals with associated dates as archival research established the source of the radiocarbon dates. The second and largest group comprised those whose dates are estimated from the age of the settlement or site, where good quality radiocarbon dates exist, where the age of the settlement is uncontroversial (based on material culture finds) and where the excavator interpreted the burials to be associated with the site chronology (*N* = 156). Finally, also included are those dates considered least secure, where the chronological information available for the site is based on incongruous or a small number of radiocarbon dates, or where information connecting the burials to the settlement is ambiguous or difficult to recover (i.e. older excavations, published in hard-to-source reports) (*N* = 74).

Kernel Density Estimation (KDE) and Summed Probability Distribution (SPD) methods were used to summarise the distributions of the ages with three models developed to progressively incorporate the data of the three confidence levels. We deployed the *KDE_Model* tool from OxCal (v. 4.4^87^), which aggregates repeated subsamples of KDEs based on sampling of the data points^88^. This results in a more conservative estimate of the distribution of dates through time over another commonly used method, the SPD, and offers a smoother curve that does not overemphasise minor features of the distribution, meaning it is more suitable for use with smaller sample sizes. Moreover, the resulting aggregate KDE provides a visual estimate of uncertainty, with an envelope around the KDE that reflects the individual KDE models. The simulated ages for the individuals in the latter two categories of data confidence were incorporated in the models using the *R_Simulate* command in OxCal, which generates a random radiocarbon age within the specified age bounds.

**Fig. 4:**
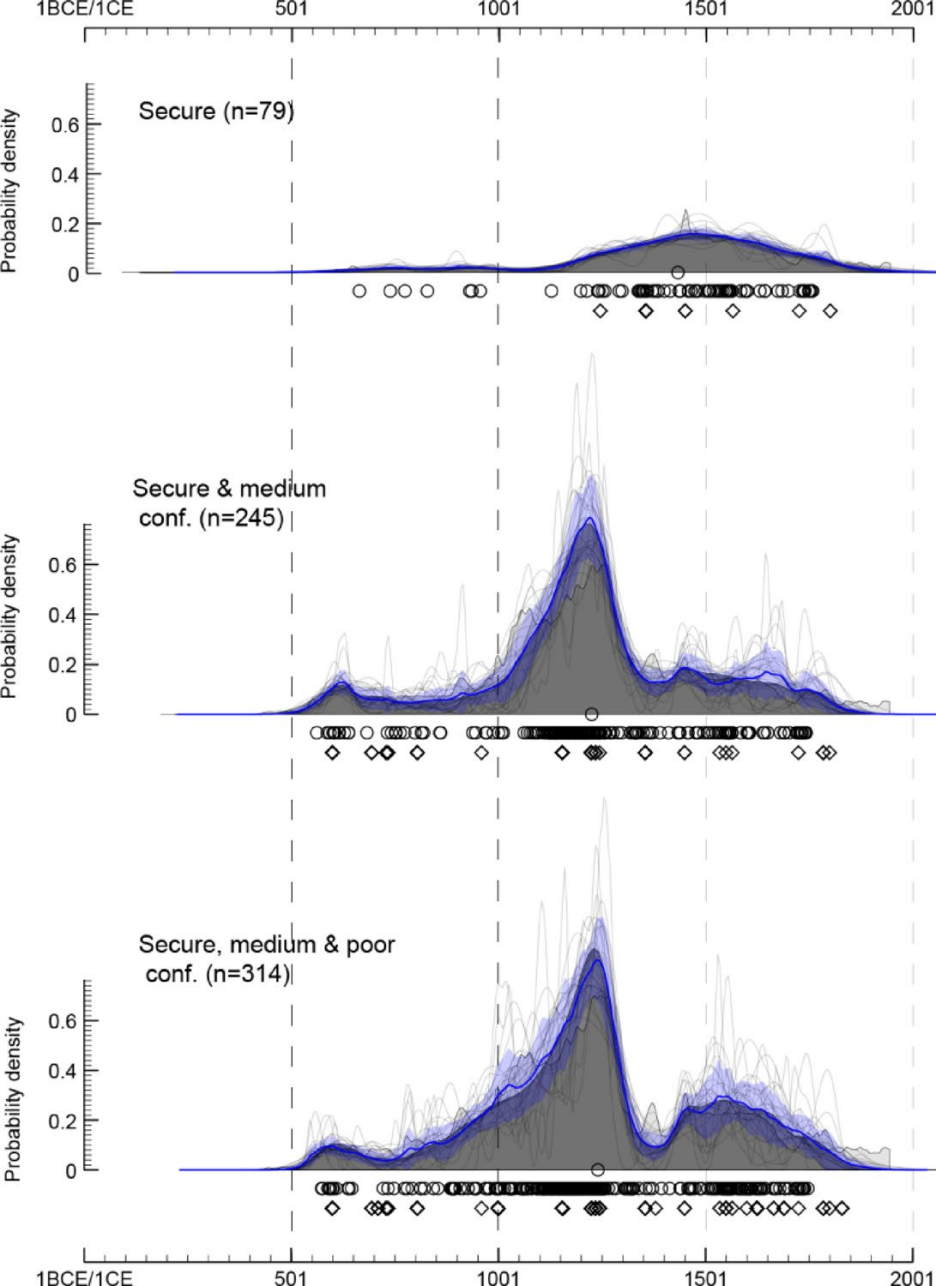
Kernel density estimation models of radiocarbon dates and simulated ages (as specified by the diamonds) that progressively incorporate data of increasingly less confidence, from top to bottom (OxCal v. 4.4, Bronk Ramsey^88^). Dates are calibrated using the southern hemisphere calibration curve SHCal20 (Hogg et al.,^91^), with no adjustments for marine reservoirs necessary.

Figure 4 shows the three KDEs, progressively incorporating the lower confidence age categories from top to bottom. The topmost KDE is broadly congruent with that published in Loftus et al.^89^, showing two approximate clusters of dates separated by a gap soon after the turn of the first millennium, but with more simulated ages in the second millennium, based on closely associated ages. This model does not reflect the known distribution of burials from archaeological sites. The second model incorporates 166 additional simulated ages and yields a remarkably different distribution of human remains through time, with a notable peak centred on c. 1200 CE. This peak reflects the 153 simulated ages from just 3 closely related sites (Schroda, K2 and Mapungubwe) attributed to the so-called Middle Iron Age phase of the archaeological sequence in the Shashe-Limpopo Basin. Importantly, this KDE far better reflects the known distribution of archaeological human remains, although the dominant peak may obscure further details of the distribution. Finally, an additional 69 simulated ages of poor confidence do not radically change the shape of the distribution, although this model has a less defined start to the peak and a possible second peak of dates in the second part of the 2nd millennium CE. This suggests that the “poor” confidence dates are broadly in line with the moderate confidence dataset, and can be incorporated into an aggregate model for archaeological interpretation.

**Fig. 5:**
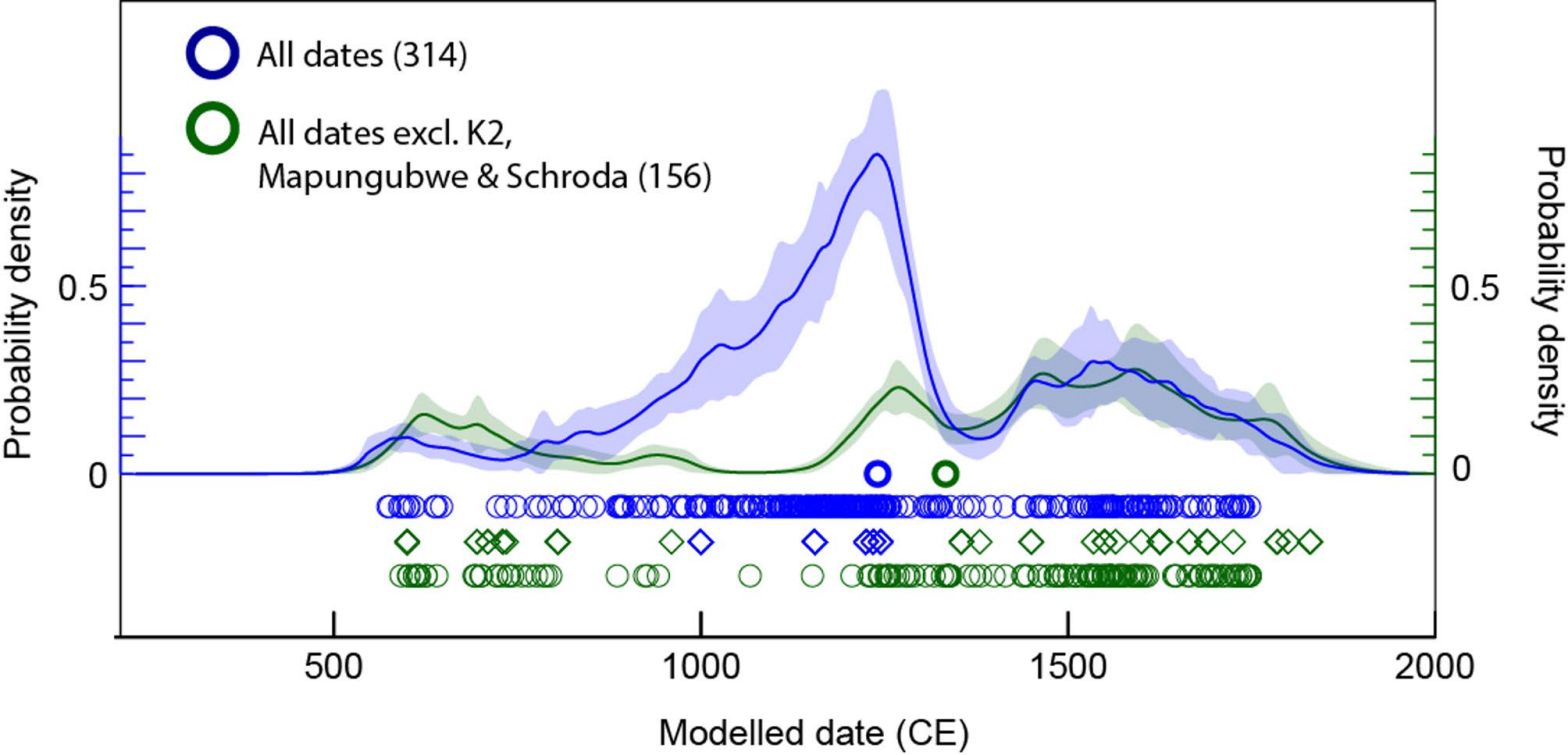
Kernel density estimation models of radiocarbon dates and simulated ages (as specified by the diamonds) for all human remains (blue) and excluding 153 simulated ages from three archaeological assemblages (Schroda, K2 and Mapungubwe) (green).

Figure 5 shows the full model, against a model that excludes 153 simulated dates from just three Shashe-Limpopo Basin Middle Iron Age sites, Schroda (*N* = 26), K2 (*N* = 101) and Mapungubwe (*N* = 25). This is to demonstrate the large impact that these three assemblages of human remains have on the national model. This model shows a marked decline over the period of the Middle Iron Age, highlighting the significance of the Shashe-Limpopo landscape over this interval. The smaller model still yields peaks in the earlier part of the curve (i.e. before 1000 CE) and after the main peak of the full model, reflecting fluctuations in the occurrence of human remains outside of the Shashe-Limpopo Basin.

**Fig. 6:**
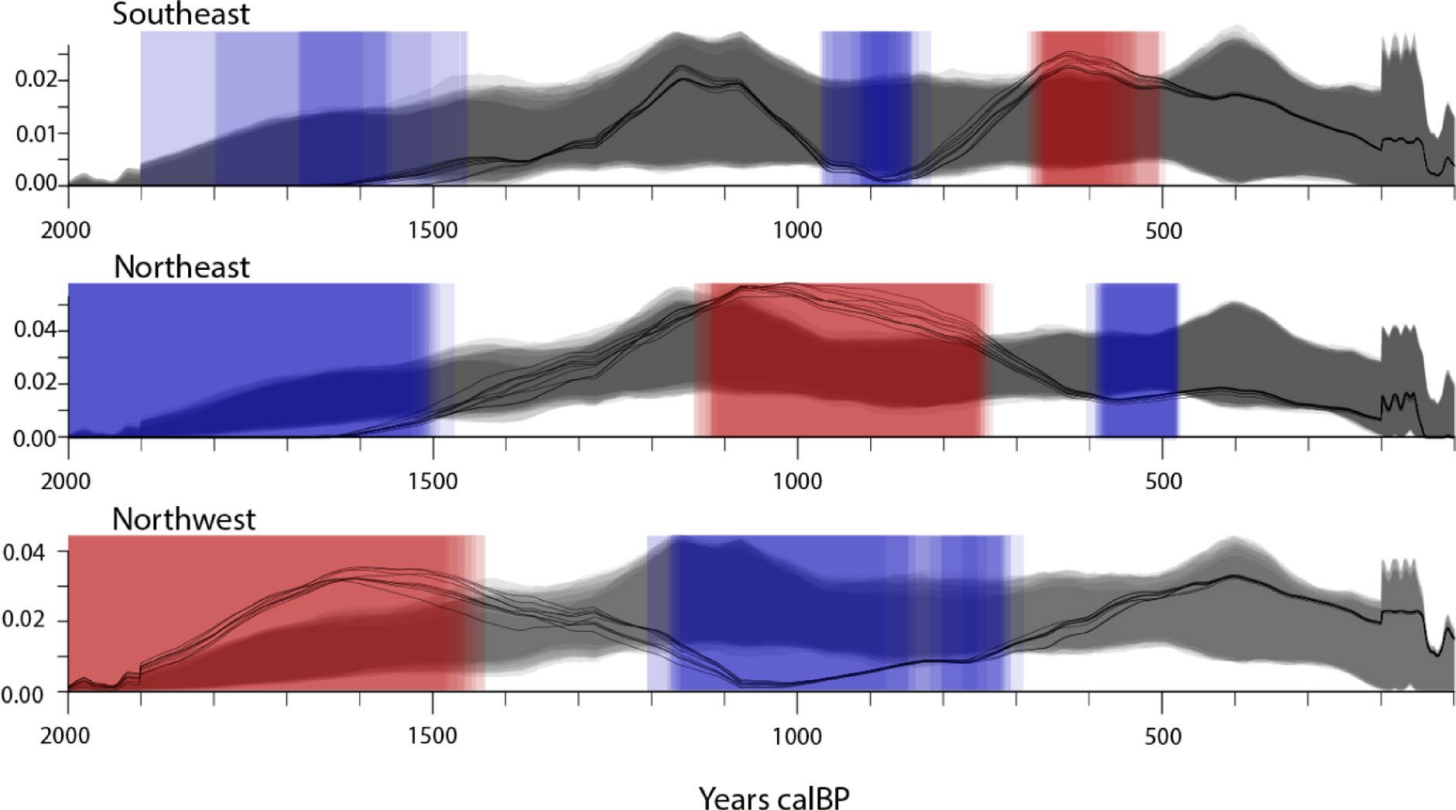
Ten overlain iterations of the permTest function in rcarbon (Crema and Bevan^89^), with updated simulations of the “Medium” and “Poor” quality sets in each iteration (using a random generator, based on the modelled age ranges).

Divided into subregions (Fig. 2), the three models display markedly different patterns of human remains frequency through time - particularly stark is the apparent antiphase relationship in the patterning of the north-east and north-west models. It may be significant, however, that the sample sizes are very different between the subsets, which presumably affects the reliability of the interpretations, particularly for the smallest model in the southeast. We evaluated differences in the regional distributions using the permTest function from rcarbon^89,90^.The non-direct burial “dates” are simulated using a random generator based on the modelled site age estimates (± 1σ). Figure 6 shows 10 replicate models for the three regions (allowing the randomly simulated dates to update between each model) overlain upon each other. Coloured areas in the plots reflect deviations from the overall distribution - note that the antiphase relationship between the NE and NW models persists across all iterations, indicating that this is not a function of sample size. Moreover, despite greater uncertainty for the smallest, SE model (as reflected in the larger grey simulation envelope), the antiphase patterning with the NE region after c. 1000 calBP is evident across all iterations, although with less stability in the precise timing of the peaks and troughs (likely reflecting the small sample).

Another important point is that the three regions are differently affected by the method used here of simulating dates for undated burials. While both the northwest and southeast groups have dates distributed more or less equally across the different confidence levels (i.e. 53% of north-west ages and 60% of south-east are in the medium and poor confidence categories with simulated ages), such data make up nearly 90% of the north-east model (due largely to the impact of the large assemblages of human remains from the Shashe-Limpopo Basin). Thus, this method has important implications for allowing us to study the patterning between regions, as well as within them.

## Electronic supplementary material

Below is the link to the electronic supplementary material.

Supplementary Material 1

## Data Availability

All of the data used to generate the burial frequency distributions, including a spreadsheet of all burials with age information and the the OxCal code for the KDE models is available at https://github.com/emmaloftus/South-African-Iron-Age-burials. Change point detection was accomplished using the findchangepts package in MATLAB, identifying where the root-mean-square of each signal changes most significantly.
